# Evaluating Oral Health Status in Incarcerated Women: A Systematic Review

**DOI:** 10.3390/jcm14051499

**Published:** 2025-02-24

**Authors:** Jakub Fiegler-Rudol, Monika Tysiąc-Miśta, Janusz Kasperczyk

**Affiliations:** 1 Student Scientific Society at the Department of Medicine and Environmental Epidemiology, Faculty of Medical Sciences in Zabrze, Medical University of Silesia, 41-800 Katowice, Poland; 2 Department of Conservative Dentistry with Endodontics, Faculty of Medical Sciences in Zabrze, Medical University of Silesia, 40-055 Katowice, Poland; mtysiac-mista@sum.edu.pl; 3 Department of Medicine and Environmental Epidemiology, Faculty of Medical Sciences in Zabrze, Medical University of Silesia, 40-055 Katowice, Poland; jkasperczyk@sum.edu.pl

**Keywords:** incarcerated women, oral health, dental caries, periodontal disease, health disparities, correctional healthcare, systemic neglect, gender-specific health

## Abstract

**Background**: Oral health is crucial to overall well-being but is significantly neglected among incarcerated women, who face higher rates of dental caries, periodontal disease, and edentulism due to systemic barriers, behavioral risks, and socio-demographic vulnerabilities. **Objective**: This review evaluates the oral health status of incarcerated women, identifying key determinants and assessing intervention effectiveness. **Methods**: A systematic search of PubMed, Scopus, Embase, and Cochrane Library databases was conducted as per PRISMA 2020 guidelines. Studies published in English in the last 15 years on adult incarcerated women were included. Data on oral health outcomes, risk factors, and interventions were extracted and analyzed. **Results**: Ten studies revealed significantly higher rates of oral diseases among incarcerated women compared to the general population. Behavioral factors such as smoking and poor diet, combined with inadequate access to care, are major contributors. Pregnant inmates face compounded risks, with poor oral health linked to adverse maternal and infant outcomes. **Conclusions**: Incarcerated women experience severe oral health disparities requiring gender-responsive, interdisciplinary interventions, including preventive care, education, and integrated correctional policies. Future research should focus on longitudinal studies and effective intervention strategies.

## 1. Introduction

Oral health is a vital component of overall well-being, yet it remains largely neglected in incarcerated populations [1]. Mounting evidence underscores the connection between poor oral health and systemic conditions such as cardiovascular disease, diabetes, and adverse pregnancy outcomes, raising significant concerns for high-risk groups [2,3,4]. Inmates frequently present with worse oral health than the general population, driven by limited access to preventative and restorative services, inadequate prison healthcare infrastructure, and a shortage of dental professionals [5]. This poor oral health status often reflects pre-incarceration disadvantages—many individuals have had minimal or no prior dental care, and some receive their first-ever dental examination upon entering prison. Compounding these challenges, environmental and psychological stressors within correctional facilities further undermine healthy oral behaviors, leading to poorer outcomes [6]. Female inmates contend with additional barriers stemming from gender-specific health concerns, histories of trauma, and inadequate access to appropriate care. Although these patterns are evident in various prison systems worldwide—including in Europe—they are especially pronounced among women, highlighting a global dimension to the issue [7]. Overall, incarcerated individuals face significant oral health disparities, including high rates of untreated dental caries, periodontal disease, and edentulism [8,9,10,11,12,13]. In many correctional facilities, dental care is restricted to acute emergencies; preventative and restorative procedures such as fillings or crowns are rarely available due to resource constraints [14]. Consequently, treatment often defaults to extractions, prompting criticisms that correctional healthcare models neglect rehabilitation and fail to provide equitable access to care [15,16]. The social determinants of health also weigh heavily on this population. Low socioeconomic status, poor health literacy, limited education, and high rates of substance use exacerbate oral health vulnerabilities [16,17]. A substantial number of incarcerated individuals enter prison with significant unmet health needs that remain largely unaddressed, perpetuating a cycle of neglect and poor long-term outcomes [18]. Women in correctional settings exemplify these challenges; they frequently arrive with poorer baseline health than men, shaped by trauma, abuse, and substance dependency, all of which contribute to severely compromised oral health [19,20]. Limited access to preventative and restorative care can lead to unchecked conditions such as persistent dental pain, rampant caries, and advanced periodontal disease [8,9,10,11,12,13,14]. For instance, in Karachi, Pakistan, 63.6% of incarcerated women who smoked exhibited moderate to severe periodontitis, illustrating the compounded effect of behavioral risks and inadequate healthcare [21]. Additionally, pregnant inmates with poor oral health face heightened risks for adverse outcomes, such as preterm birth and low birth weight [22]. Beyond immediate physical consequences, neglected oral health damages self-esteem and amplifies the social stigma of incarceration, impeding effective rehabilitation and reintegration [13]. Given these compounded challenges, especially among female inmates, targeted interventions and more robust research are urgently needed to break the cycle of neglect and ensure equitable oral healthcare for those in correctional facilities.

### Objectives

This systematic review examines the oral health of incarcerated women, including caries, periodontal disease, and mucosal pathologies, while identifying key behavioral, systemic, and environmental determinants. It evaluates global oral health interventions in women’s prisons and provides evidence-based, gender-responsive recommendations to improve preventative care, inform policy, and reduce oral health inequities.

## 2. Materials and Methods

### 2.1. Focused Question

This systematic review was conducted using the PICO framework [23], as shown in Table 1.

### 2.2. Search Strategy

**Figure 1:**
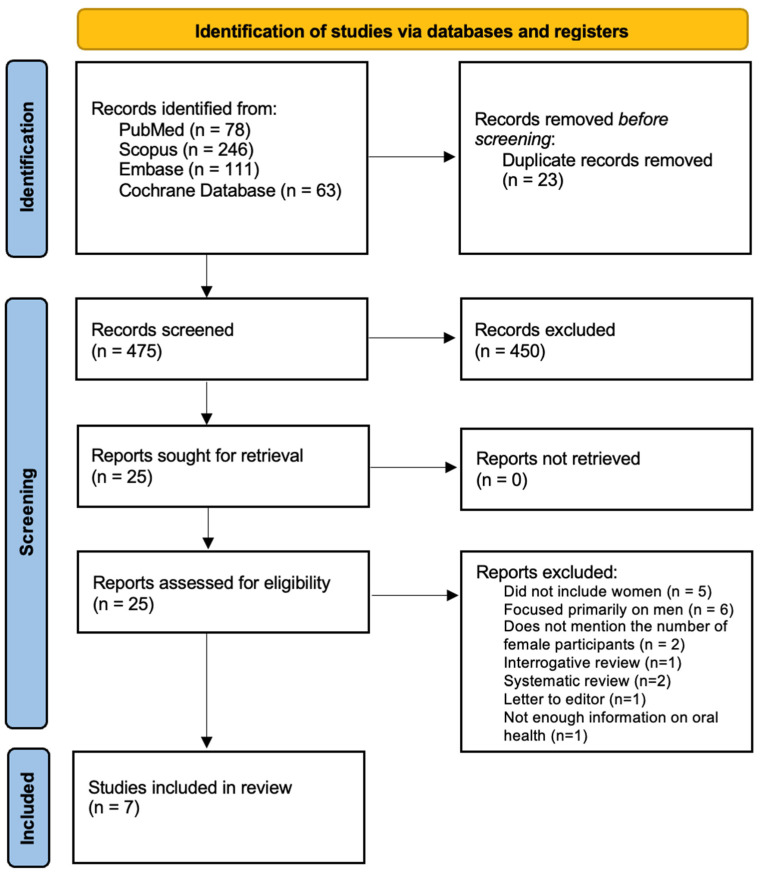
Prisma 2020 flow diagram.

This article was registered under PROSPERO with the registration number CRD42024621672. This systematic review adhered to the PRISMA 2020 guidelines [24]. An electronic search was independently conducted by three authors across PubMed/Medline, Scopus, Embase, and Cochrane Library databases (search terms provided in Figure 1). Filters were applied to include only articles published between in the past 15 years and limited to English-language publications. Following an initial screening, studies were selected based on titles and abstracts to assess their eligibility against the inclusion criteria. The search syntax for each database is given in Table 2. The authors then performed a detailed full-text review of the selected studies to gather relevant data.

### 2.3. Study Selection

This research proposed that incarcerated women may exhibit a higher prevalence of oral health issues, such as dental caries, periodontal diseases, mucosal pathologies, and poor oral hygiene, potentially highlighting the need for targeted oral health interventions in this population. Articles included in this review were selected based on well-defined inclusion and exclusion criteria. During the study selection process for this systematic review, reviewers independently evaluated the titles and abstracts of the identified articles to minimize bias. Any disagreements regarding the eligibility of studies were resolved through collaborative discussions until a unanimous decision was achieved. This rigorous methodology, adhering to PRISMA guidelines, helped ensure that only the most relevant and high-quality studies were included in the analysis, strengthening the review’s reliability and reproducibility.

Inclusion Criteria:

- Articles published in English
- Articles published in the past 15 years.
- Studies examining the oral health of adult inmates aged 18 and older
- Only studies that account for the duration of inmates’ incarceration, as longer stays may affect oral health outcomes.
- Only studies with specific research designs, such as cross-sectional, cohort, or randomized controlled trials, to ensure high-quality data and relevance.

Exclusion Criteria:

- Non-peer-reviewed sources.
- Studies focused on the oral health of inmates under 18 years old.
- Study Types: case reports or case series, letters to the editor, historical overviews, narrative or systematic reviews, books, documents, and other non-journal sources.
- “Gray literature” sources.
- Duplicate publications or studies sharing the same ethical approval number.
- Studies published in languages other than English.
- Studies that do not report specific oral health outcomes or lack objective oral health assessments.
- Data from non-correctional settings.
- Non-original research: commentaries, editorials, and opinion pieces.

### 2.4. Risk of Bias in Individual Studies

During the preliminary stage of study selection, reviewers independently examined titles and abstracts to minimize potential bias in the evaluation. Inter-reviewer agreement was measured using Cohen’s kappa statistics to ensure consistency in decision-making [25]. Disagreements concerning the inclusion or exclusion of studies were resolved through thorough discussions among the authors until a unanimous decision was achieved. Moreover, the potential impact of hormonal changes, pregnancy, and other gender-specific factors on oral health were considered during the review, and variations in study methodologies were assessed to ensure a comprehensive and accurate synthesis of findings.

### 2.5. Quality Assessment

Two reviewers (J.F.-R. and M.T.-M.) independently screened the included studies to assess their quality. The evaluation criteria focused on key aspects of study design, implementation, and analysis related to oral health outcomes in incarcerated women, emphasizing objectivity and verification of results. The risk of bias was determined by the number of “yes” or “no” responses assigned to each study based on the following questions:

1. Was the study design appropriate for addressing the research question?
2. Were the inclusion and exclusion criteria clearly defined?
3. Was the sample size adequate?
4. Was the selection of participants free from bias?
5. Were the oral health outcomes measured using valid and reliable methods?
6. Were the examiners calibrated and blinded to the participants’ incarceration status?
7. Was a non-incarcerated control group included in the study?
8. Were potential confounding factors identified and controlled for?
9. Were numerical results reported with relevant statistics?
10. Was there no missing outcome data affecting the results?

The information collected from each study was assessed, and a classification was applied based on the total number of “yes” answers to the above questions. The degree of bias was calculated according to the following point limits:

1. High risk of bias: 0–3 points
2. Moderate risk of bias: 4–6 points
3. Low risk of bias: 7–10 points

The scores for each study were calculated, and an overall estimated risk of bias (low, moderate, or high) was assigned to each included study, following the recommendations outlined in the Cochrane Handbook for Systematic Reviews of Interventions [26].

Table 3 presents the risk of bias evaluations for the seven studies selected after the full-text review. Each study had to achieve at least 6 points to be included in the analysis. All of the included studies were found to have a low risk of bias, with four of them receiving the highest possible rating of 9. None of the studies were categorized as moderate or high risk.

To evaluate the methodological rigor of the included studies, we adapted our quality assessment from established guidelines in the Cochrane Handbook for Systematic Reviews of Interventions, which recommend examining key domains such as study design, participant selection, outcome assessment, and data reporting [26]. This approach ensures a transparent, systematic evaluation of potential sources of bias. Our use of a ten-item checklist is consistent with these recommendations, allowing us to gauge risk of bias levels by focusing on core features—like clear inclusion criteria, reliable outcome measures, and appropriate control of confounding factors. Each item was designed to reflect internationally recognized standards for epidemiological research, particularly in vulnerable and hard-to-reach populations such as incarcerated individuals. Assigning “yes” or “no” responses across these items helps stratify studies into low, moderate, or high risk of bias, ensuring a more uniform appraisal that supports evidence-based conclusions.

### 2.6. Data Extraction

After reaching a consensus on the selection of included articles, the three reviewers extracted data on various aspects relevant to the study objectives. This included citation details (first author and publication year), type of study, number of participants, and age range or mean age. They also recorded the duration of incarceration, ethnicity, and socioeconomic status (if available), along with descriptions of the incarcerated women group and, where applicable, the non-incarcerated control group. For longitudinal studies, the duration of follow-up was noted. Data on oral health conditions, such as dental caries, periodontal diseases, mucosal pathologies, and oral hygiene status, were extracted, along with prevalence and incidence rates, the severity of oral health issues, and the diagnostic criteria and indices used (e.g., DMFT index, CPI). Examination procedures, tools, and behaviors such as tobacco use, alcohol consumption, and substance abuse were also included. Additional factors assessed were oral hygiene practices, dietary habits, access to dental care services, details of any oral health interventions, and education programs or preventive measures implemented. Statistical findings, significance levels, and comparisons between incarcerated and non-incarcerated groups (if applicable) were documented. The authors’ main conclusions, suggestions for clinical practice or policy changes, acknowledged limitations, sources of funding, and any declared conflicts of interest were also recorded to ensure a comprehensive analysis of the selected studies.

## 3. Results

### 3.1. Study Selection

Figure 1 provides a comprehensive overview of the study selection process, following PRISMA guidelines [23]. An initial database search retrieved 498 articles; after removing duplicates, 455 remained. Screening titles and abstracts narrowed the pool to 28 studies for full-text evaluation. Of these, twenty-one were excluded for the following reasons: five did not include female participants, six focused predominantly on men (thus preventing separate conclusions for women), two omitted the number of female patients, 1 was an interrogative review, two were systematic reviews, one was a letter to the editor, and one lacked information on oral health. Consequently, seven studies published within the past decade were retained for the final review. A summary of these studies is provided in Table 3.

### 3.2. Data Presentation

The extracted data from the seven studies that met the eligibility criteria and were included in the review are summarized in Table 4, Table 5, Table 6 and Table 7.

### 3.3. General Characteristics of the Included Studies

**Table 4:** A general overview of the studies.

First Author	Year	Country	Study Period	Study Design	Patients (*n*)	Female%	Mean Age
George et al. [8]	2012	India	April–May 2009	Cross-sectional	1060	3.3	34.6
Konratyev et al. [9]	2019	Russia	2017–2018	Cross-sectional	305	42.6	33.0 ± 5
Rodrigues et al. [11]	2014	Brazil	Not specified	Cross-sectional	65	100	32.2 ± 11.56
Rouxel et al. [12]	2013	UK	July–August 2010	Cross-sectional	103	100	30.9 ± 9.6
Soares et al. [13]	2019	Brazil	June and December 2015	Cross-sectional	305	100	32.1
Shah et al. [21]	2023	Pakistan	December 2021–February 2022	Cross-sectional	131	100	34.73 ± 9.94
Testa et al. [22]	2022	USA	2016–2019	Logistic regression	Not specified	Not specified	Not specified

### 3.4. Main Study Outcomes

Research on oral health among incarcerated populations has consistently demonstrated widespread disparities, significant unmet needs, and alarming deficiencies in dental care access, all of which severely impact the quality of life and overall well-being of this vulnerable group [8,9,10,11,12,13,14,15,16,17,18,19,20,21,22]. High rates of dental caries, ranging from 75% to 92.4%, are a pervasive issue, with a substantial proportion of prisoners suffering from untreated decay, missing teeth, and periodontal diseases, including gingivitis and periodontitis, which affect up to 55.7% of individuals in some populations [9,11,12]. These oral health challenges are compounded by additional conditions such as diseases of the pulp and periapical tissues, cheilitis, glossitis, and smoking-related lesions, which further degrade oral health status [9,21]. Tooth loss is particularly widespread, with many inmates experiencing severe edentulism and requiring prosthetic rehabilitation due to the absence of adequate dental interventions [8,11]. Factors contributing to poor oral health outcomes in prisons include behavioral and socio-demographic elements such as high smoking prevalence, low levels of education, poor dietary habits (e.g., high sugar intake), prior drug use, and longer durations of incarceration [9,12,21]. These risk factors not only exacerbate oral health problems but also significantly limit prisoners’ ability to maintain oral hygiene or access preventive and therapeutic dental care. Many prisoners report severe barriers to accessing dental services, with nearly all citing long waiting lists, infrequent dentist availability, and systemic inadequacies as major obstacles [12]. Despite a reported willingness to seek care, the lack of timely and appropriate dental interventions often results in the progression of oral diseases, increased pain, and diminished oral function, further impairing the quality of life [12,13]. Oral health-related quality of life (OHRQoL) is severely impacted in this population, as studies consistently show that issues like dental caries, tooth loss, and periodontal diseases correlate with significant physical pain, psychological discomfort, and functional impairments [13,22]. Many inmates report difficulty eating, speaking, and performing routine tasks, with conditions such as dental pain and prosthetic needs increasing the likelihood of these impairments by more than threefold [13]. Furthermore, the mental and emotional toll of poor oral health adds another layer of disadvantage to this population, as their oral health status often serves as a barrier to social reintegration and overall well-being [22]. Particularly vulnerable groups, such as female inmates, pregnant women, and older prisoners, face disproportionately higher oral health challenges. Studies have shown that incarcerated women, especially those who smoke, experience severe periodontitis at higher rates, while pregnant women in prison are burdened with poor oral health, inadequate healthcare knowledge, and increased risks of adverse maternal and infant outcomes due to unmet oral health needs [21,22]. The situation is further exacerbated by the lack of tailored interventions and the absence of integrated healthcare frameworks within prison settings, which leave these subgroups at heightened risk of both oral and systemic health complications [22]. Importantly, many individuals enter the correctional system with pre-existing, untreated oral health conditions; for some, incarceration is the first time they receive any form of professional dental care, reflecting broader societal inequities rather than solely prison-based issues [13,14,15,16,17,18,19,20,21]. Moreover, there are substantial differences between countries regarding prison healthcare policies, resource allocation, and cultural factors, highlighting the importance of comparative international research to fully understand the scope and variability of oral health outcomes among incarcerated populations [21,22,23].

**Table 5:** An overview of the main study outcomes.

Author and Year	Main Study Outcomes
George et al. [8]	Among women, 8.6% had a partial denture in the maxilla, and 5.7% had one in the mandibule. Edentulousness was prevalent, underscoring the need for prosthodontic services within prisons to address tooth loss and improve inmates’ oral health. The study recommends integrating oral healthcare facilities in prison settings to prevent disease progression and enhance the quality of life for this disadvantaged group.
Konratyev et al. [9]	Complete dentition = 10.5%, partial tooth loss = 81.9%, edentulism = 7.5%. Dental caries = 92.4%, periodontitis = 55.7%, gingivitis = 33.1%, diseases of the pulp and periapical tissue = 87.9%. Cheilitis = 64.9%, glossitis = 21.9%, palatal nicotinic leukokeratosis = 28.8%. OHI-S and DMFT indices were strongly associated with incarceration duration and education level, with higher scores in inmates incarcerated longer and with lower education (*p* = 0.001).
Rodrigues et al. [11]	The mean number of missing teeth was 11.3. A significant association was found between tooth loss and oral health satisfaction (*p* = 0.049), self-perceived need for dental prostheses (*p* < 0.001), discomfort during toothbrushing (*p* = 0.005), difficulty speaking (*p* = 0.002), and challenges in performing routine tasks (*p* = 0.025). It was noted that 29.2% of inmates used some form of prosthesis, all of which were unsuitable, while 78.5% required prosthetic rehabilitation. The oral health condition of the studied population was poor, characterized by significant tooth loss and a high need for dentures, with tooth extraction being the primary reason for seeking dental care.
Rouxel et al. [12]	Dental health metrics showed worse conditions than the 2009 ADHS benchmarks, with higher levels of decay (mean DMFT-score 12.3 vs. 11.4), untreated dental caries (75% vs. 39%), and periodontal issues (60% had pocketing ≥ 4 mm vs. 41%). Smoking (66% vs. 26%), high sugar intake (66%), and prior drug use (50%) were prevalent. Most prisoners (97%) reported difficulty accessing prison dental services, citing long waiting lists and infrequent dentist availability. Oral health impacts, including pain and functional impairments, were reported by 73%, double the ADHS rate. Despite most prisoners brushing teeth twice daily and having prior dental visits, dental care access and outcomes in prison remained critical concerns.
Soares et al. [13]	Dental issues were prevalent, with a mean DMFT score of 11.7 ± 6.33, predominantly driven by decayed teeth in younger inmates and missing teeth in older ones. Nearly 84% had at least one carious lesion, and only 2.6% exhibited fully healthy periodontal sextants, with calculus being the most common periodontal issue (38.7%). Over half (57.2%) experienced dental pain in the prior six months, and 91% perceived a need for dental care. Oral health significantly impacted quality of life (mean OHIP-14 score 19.16 ± 14.53), with “physical pain” and “psychological discomfort” being the most affected dimensions. Dental caries, tooth loss, periodontal pockets, prosthodontic needs, and dental pain were strongly associated with lower oral health-related quality of life (OHRQoL). Inmates with dental caries or pain were 3.64 and 4.04 times more likely, respectively, to report oral health impacts. Older inmates and those with prosthodontic needs exhibited significantly higher OHRQoL scores, highlighting the critical need for improved dental care and education in this population.
Shah et al. [21]	76 (58%) of women had a DMFT score of 0–5, 42 (32.1%) scored between 6 and 10, and 13 (9.9%) scored 11 or higher. Among smokers, 14 (63.6%) and 2 (9.1%) had moderate to severe periodontitis, respectively, with a statistically significant association between smoking and severe periodontitis (*p* = 0.023). Additionally, employment status prior to incarceration was significantly linked to smoking (*p* = 0.02). The prevalence of severe periodontitis was notably higher among incarcerated women who smoked. Regular oral health assessments and increased education on oral health management are essential for women in prison. However, smoking did not show a significant association with dental caries or general periodontal disease.
Testa et al. [22]	Women who experience incarceration during pregnancy have poorer oral health, limited healthcare knowledge, and greater unmet oral healthcare needs. Given the heightened risks of adverse maternal and infant health outcomes in this population, as well as the role of poor oral health in pregnancy complications and negative birth outcomes, the findings underscore the need for collaboration among experts in criminal justice, public health, and oral health. Such interdisciplinary efforts are essential to advancing health equity for pregnant women impacted by incarceration.

ADHS—Adult Dental Health Survey; DMFT: D: Decayed teeth M: Missing teeth due to caries F: Filled teeth.

### 3.5. Hard Tissue Conditions

**Table 6:** Summary of hard tissue conditions in studies.

Study	Prevalence of Caries	Mean DMFT	Decayed Teeth (D)	Missing Teeth (M)	Filled Teeth (F)
Konratyev et al. [9]	92.4%	14.86 ± 0.26	6.48 ± 2.18	2.92 ± 1.24	4.24 ± 1.29
Rodrigues et al. [11]	84%	20.37 ± 7.87	7.83 ± 5.01	11.26 ± 10.38	1.28 ± 2.58
Rouxel et al. [12]	75%	12.30 ± 7.48	2.47 ± 2.52	4.96 ± 4.92	4.87 ± 4.68
Soares et al. [13]	84%	11.7 ± 6.33	3.8 ± 3.46	4.4 ± 6.29	3.5 ± 3.53
Shah et al. [21]	58% (DMFT 0–5), 32.1% (DMFT 6–10), 9.9% (DMFT ≥ 11)				

### 3.6. Periodontal Tissue Conditions

**Table 7:** Summary of Periodontal conditions in studies.

Study	Periodontal Results
Konratyev et al. [9]	Periodontitis = 55.7%, gingivitis = 33.1%, diseases of the pulp and periapical tissue = 87.9%. Cheilitis = 64.9%, glossitis = 21.9%, palatal nicotinic leukokeratosis = 28.8%.
Rouxel et al. [12]	Periodontal issues (60% had pocketing ≥ 4 mm vs. 41%). Smoking (66% vs. 26%), high sugar intake (66%), and prior drug use (50%) were prevalent.
Shah et al. [21]	Among smokers, 14 (63.6%) and 2 (9.1%) had moderate to severe periodontitis, respectively, with a statistically significant association between smoking and severe periodontitis (*p* = 0.023). The prevalence of severe periodontitis was notably higher among incarcerated women who smoked. Regular oral health assessments and increased education on oral health management are essential for women in prison.
Soares et al. [13]	The most common issue among the examined women was calculus, affecting 38.7% of cases. This was followed by the presence of shallow pockets (26.2%), excluded sextants (17.7%), deep pockets (11.5%), and bleeding on probing (3.3%). Only 2.6% of individuals had all sextants in a healthy state.

## 4. Discussion

### 4.1. Results in the Context of Other Evidence

The evidence considered in this systematic review indicated that we should reject our null hypothesis. Incarcerated women have significantly poorer oral health compared to the general population, with a high prevalence of dental caries, periodontal diseases, and edentulism [8,9,11,12,13]. This is in line with other studies in this area, which showed that female prisoners demonstrated worse oral health than the general population, characterized by elevated levels of oral diseases, infections, and substantial dental treatment needs [27,28,29,30,31]. A study focused exclusively on female prisoners by Rouxel et al. highlighted significant oral health challenges and a high prevalence of unmet dental care needs within this population [12]. Factors such as smoking, substance dependency, poor diet, and low health literacy contribute to the severity of oral health issues in this population [9,12,13,21]. This is in line with the current medical literature [32,33,34]. Limited access to preventative and restorative dental care is a systemic problem in correctional facilities. Most care is reactive, focusing on extractions rather than comprehensive treatments [8,11,12]. This trend exists in the prison population assessed in other studies [18,35,36]. Studies showed a greater incidence of caries in incarcerated women, compared to the general population [9,11,12,13,21]. O’Hara et al. attribute the high rate of caries in the prison population to their greater propensity for engaging in health-damaging behaviors, adherence to a highly cariogenic diet, and inadequate oral hygiene practices [37]. Poor oral health has profound psychosocial consequences, including diminished self-esteem and impaired ability to reintegrate into society post-incarceration, which is a correlation observed in the general population as well [11,13,38]. Incarcerated pregnant women face unique challenges, with poor oral health linked to adverse maternal and infant health outcomes, such as preterm births and low birth weights [22]. Female inmates often have histories of trauma and abuse, which exacerbate their oral health vulnerabilities. These issues are compounded by systemic neglect within prison healthcare systems [9,21,22]. There is a significant unmet need for educational and preventive oral health interventions, including regular assessments and targeted health promotion [12,13,21]. Longer incarceration periods and lower education levels are associated with worse oral health outcomes, as indicated by higher DMFT (Decayed, Missing, and Filled Teeth) scores [9,11,13]. Smoking is a critical risk factor for severe periodontitis among incarcerated women, necessitating focused interventions for smoking cessation and oral health education [21]. Other studies demonstrated similar results. Treadwell et al. highlighted significant service and policy gaps in Georgia’s oral health network, which hinder the ability of incarcerated women and those re-entering society to address their oral care needs [39]. Moraes et al. revealed that oral health significantly impacts the self-perceived quality of life of incarcerated women in Minas Gerais, Brazil [40]. Bull et al. concluded that the complexity of prisoners’ oral health is influenced by both individual and systemic factors, underscoring the need for further studies to address gaps in the literature [1]. Longhi et al. concluded that the incarceration of women, compounded by inadequate prison healthcare, significantly impacts their oral health and well-being, highlighting an urgent need for comprehensive research and collaborative health interventions to improve their conditions [41]. Yang et al. reported that oral, salivary gland, and jaw diseases were more prevalent among female prisoners compared to their male counterparts [42]. Furthermore, the World Health Organisation reported that research on individuals involved with the criminal justice system has revealed the following: a significant prevalence of dental caries and periodontal disease [43]; a high incidence of tooth decay, ranging from 57% to 67% [44,45]; inadequate oral health and dietary practices [46]; compromised oral health-related quality of life, negatively affecting daily functioning [12,47,48]. Nevertheless, it is important to recognize that many individuals who are at risk of incarceration already display higher baseline rates of oral health problems [47]. Socioeconomic disadvantages, prior lack of access to dental care, and other behavioral factors may predispose these individuals to poor oral health before entering prison. Consequently, comparing incarcerated women solely to the broader general population may overlook significant differences in risk profiles between these groups [48].

In the general female population, DMFT scores consistently appear lower than those reported among incarcerated female populations. A study reported a mean DMFT score of 5.31 ± 5.79 among adult females aged 30–34, 35–44, and 50+, indicating a relatively higher prevalence of dental caries compared to other demographics [49]. Another study found a mean DMFT of 4.63 across various age categories, with significant variations linked to education level and brushing habits [50]. In Nigeria, a study reported a notably low mean DMFT of 0.67 ± 1.6 among adult females, indicating a lower prevalence of dental caries compared to other regions, contrasting sharply with higher scores observed elsewhere [51]. Additionally, dietary habits significantly influenced DMFT scores, with one report showing a mean DMFT of 10.7 for females aged 20 to 64 years [50].

An additional oral health concern that warrants attention in this population is bruxism, which can manifest as both sleep bruxism and awake bruxism. Stress—a well-documented risk factor in incarcerated environments—significantly contributes to the onset and severity of bruxism [51,52,53,54]. Chronic bruxism often leads to mucosal maceration and can exacerbate existing oral pathologies by causing dental wear, temporomandibular joint disorders, and heightened facial muscle tension. Epidemiological data suggest that one in four individuals may experience awake bruxism overall, and among women specifically, 12% experience sleep bruxism, while 17% exhibit awake bruxism [55]. These figures underscore a potentially heightened prevalence in female populations, further reinforcing the need for gender-focused research and interventions [55]. Given the added psychosocial stressors in correctional settings, it is plausible that bruxism—and its associated complications—could be more pronounced among incarcerated women [56]. Addressing bruxism through stress management initiatives, routine screenings, and targeted interventions (e.g., occlusal splints or behavioral therapy) may be critical for improving oral health outcomes in this vulnerable group [55,56,57].

### 4.2. Limitations of the Evidence

Most of the research relies on cross-sectional studies, which, while valuable for identifying prevalence and associations, fail to establish causal relationships between incarceration and oral health outcomes. This lack of longitudinal data significantly hampers the ability to track the progression of oral health conditions over time or evaluate the long-term effectiveness of interventions. Additionally, the evidence suffers from variability in quality and methodological rigor. Many studies use convenience sampling, which introduces selection bias and limits the generalizability of findings to broader populations. The frequent absence of standardized diagnostic tools and reporting methods further complicates the synthesis of data, leading to potential inconsistencies in conclusions. Moreover, studies often prioritize descriptive over analytical approaches, providing limited insights into the multifactorial nature of oral health disparities in incarcerated women. The geographic focus of existing evidence is another critical limitation. A substantial portion of the studies is concentrated in specific regions, such as the United States and Brazil, while data from European and other global prison systems are scarce. This uneven distribution limits the applicability of findings across different sociocultural and systemic contexts, where variations in prison healthcare policies and practices could significantly influence oral health outcomes. Lastly, the evidence base lacks depth in addressing gender-specific and intersectional factors, such as the compounded effects of trauma, substance use, or pregnancy on oral health. These gaps highlight the need for more robust, comprehensive, and gender-sensitive research to inform policies and interventions tailored to the unique needs of incarcerated women.

### 4.3. Limitations of the Review Process

The limitations of this systematic review are rooted in the inherent challenges of synthesizing research from a field characterized by sparse and fragmented studies. A primary issue is the significant lack of longitudinal research examining the long-term oral health outcomes of incarcerated women, particularly studies that track their oral health during and after incarceration. This makes it difficult to establish causal relationships between incarceration-related factors, such as systemic neglect or specific health behaviors, and oral health outcomes. Another limitation is the variability in methodological quality across studies. Many studies relied on cross-sectional designs and descriptive analyses, which are insufficient to explore complex, multifactorial relationships. Additionally, the lack of standardized diagnostic criteria and consistent reporting across studies complicates the aggregation and comparison of results.

### 4.4. Implications for Practice, Policy, and Future Research

These findings underscore the urgent need for comprehensive, interdisciplinary approaches addressing prison oral health disparities. Implementing preventive and restorative dental care programs, improving accessibility to dental services, and providing targeted education on oral health management are critical steps to mitigate the burden of oral diseases in this population [8,13,22]. Such measures would enhance oral health outcomes and improve incarcerated individuals’ overall health, functionality, and quality of life. Addressing these systemic inequities is essential for promoting health equity, reducing the progression of preventable diseases, and ensuring that oral healthcare becomes an integral component of correctional health systems worldwide [12,22]. More randomized control trials and multicentre studies must be performed to explore this topic.

## 5. Conclusions

Incarcerated women face significant oral health disparities, including high rates of dental caries, periodontal disease, edentulism, and unmet treatment needs. These challenges stem from systemic barriers—limited preventive and restorative care, inadequate education, and a focus on emergency rather than comprehensive treatment—exacerbated by factors such as smoking, poor diet, and low educational attainment. Vulnerable groups, including pregnant inmates, are at even greater risk. Gender-sensitive, comprehensive interventions are urgently needed, encompassing preventive measures, routine screenings, smoking cessation, and targeted education. Integrating oral health into broader correctional policies through interdisciplinary collaboration is also critical. Future research should include longitudinal and randomized trials, expanded geographic scope, and standardized methods to establish causality and strengthen findings. Addressing these gaps will improve oral health outcomes, rehabilitation, and reintegration for incarcerated women.

## Figures and Tables

**Table 1:** The focused question for this study.

PICO Framework Component	Description
Population (P)	Incarcerated women
Intervention/Exposure (I)	Higher risk of developing oral health issues
Comparison (C)	No Direct comparison; General female population discussed in Section 4.1.
Outcome (O)	Development of oral health issues, including dental caries, periodontal diseases, mucosal pathologies, or poor oral hygiene

**Table 2:** Search syntax used across databases.

Source	Search Term	Filters	Number of Results
PubMed	(“prison” OR “incarceration” OR “inmates” OR “correctional facility” OR detainees OR imprisoned OR jail OR penitentiary OR confined) AND (“oral health” OR “oral hygiene” OR “oral status” OR “dental care” OR “dental health” OR caries OR gingivitis OR “periodontal disease” OR “dental decay” OR “oral diseases” OR “gum disease”)	English language Publication years: 2014–2024	78
Scopus	TITLE-ABS-KEY ({prison} OR {incarceration} OR {inmates} OR {correctional facility} OR {detainees} OR {imprisoned} OR {jail} OR {penitentiary} OR {correctional institution} OR {detention center} AND {oral health} OR {oral hygiene} OR {oral status} OR {dental care} OR {dental health} OR {caries} OR {gingivitis} OR {periodontal disease} OR {dental decay} OR {oral diseases} OR {gum disease} OR {tooth decay} OR {oral infection} OR {oral pathology} OR {dental plaque} OR {oral condition}) AND (LIMIT-TO (DOCTYPE, “ar”))	Article Publication years: 2014–2024	246
Embase	(‘prison’/exp OR prison OR ‘incarceration’/exp OR incarceration OR ‘inmates’/exp OR inmates OR ‘correctional facility’/exp OR ‘correctional facility’ OR detainees OR imprisoned OR ‘jail’/exp OR jail OR ‘penitentiary’/exp OR penitentiary OR confined) AND (‘oral health’/exp OR ‘oral health’ OR ‘oral hygiene’/exp OR ‘oral hygiene’ OR ‘oral status’ OR ‘dental care’/exp OR ‘dental care’ OR ‘dental health’/exp OR ‘dental health’ OR ‘caries’/exp OR caries OR ‘gingivitis’/exp OR gingivitis OR ‘periodontal disease’/exp OR ‘periodontal disease’ OR ‘dental decay’/exp OR ‘dental decay’ OR ‘oral diseases’ OR ‘gum disease’/exp OR ‘gum disease’) AND ([controlled clinical trial]/lim OR [randomized controlled trial]/lim) AND [2014–2024]/py	Publication years: 2014–2024 Controlled Clinical Trial Randomized Controlled Trial	111
Cochrane database	((mh “Prison” OR “Incarceration” OR “Inmates” OR “Correctional Facility” OR “Detainees” OR “Imprisoned” OR “Jail” OR “Penitentiary” OR “Confined” OR “Detained”) AND (mh “Oral Health” OR “Oral Hygiene” OR “Oral Status” OR “Dental Care” OR “Dental Health” OR “Caries” OR “Gingivitis” OR “Periodontal Disease” OR “Dental Decay” OR “Oral Diseases” OR “Gum Disease”))		63

**Table 3:** The results of the quality assessment and risk of bias across the studies.

Study	1	2	3	4	5	6	7	8	9	10	Score	Risk of Bias
George et al. [8]	1	1	1	0	1	0	0	0	1	1	6	Moderate
Konratyev et al. [9]	1	1	1	1	1	1	0	1	1	1	9	Low
Rodrigues et al. [11]	1	1	1	1	1	1	0	1	1	1	9	Low
Rouxel et al. [12]	1	1	1	1	1	1	1	1	1	1	10	Low
Soares et al. [13]	1	1	1	0	1	0	0	1	1	1	7	Low
Shah et al. [21]	1	1	0	1	1	1	0	0	1	1	7	Low
Testa et al. [22]	1	1	1	0	1	0	0	0	1	1	6	Moderate

## Data Availability

Not applicable.
